# Investigation of hazelnut shells driven hard carbons as anode for sodium-ion batteries produced by hydrothermal carbonization method

**DOI:** 10.55730/1300-0527.3312

**Published:** 2021-11-22

**Authors:** Elif CANBAZ, Meral AYDIN, Rezan DEMİR-ÇAKAN

**Affiliations:** Department of Chemical Engineering, Gebze Technical University, Kocaeli, Turkey

**Keywords:** Hard carbon, hydrothermal carbonization, sodium ion batteries, bio-waste

## Abstract

To be used as Na-ion battery anodes, hard carbon electrodes are synthesized from biomass, explicitly hazelnut shell (HS): via hydrothermal carbonization (HTC) followed by further pyrolysis at different temperatures (500, 750, 1000 °C). Then, the resulting hazelnut shell-based hard carbons are investigated using various methods including Fourier-transform infrared spectroscopy, scanning electron microscope, X-ray diffraction, galvanostatic charge/discharge, and electrochemical impedance spectroscopy. The effects of binders (PVdF, Na-alginate, CMC, and PAA) on electrochemical performance are determined. The obtained composite electrodes with different binders are tested in sodium half-cell configurations. A strong correlation is recognized between carbonization temperature and electrochemical performances and structural characteristics. The better cycling performance is accomplished with the electrode carbonized at 1000 °C with Na-alginate binder. After 100 cycles, specific capacity of 232 mAh × g^−1^ at 0.1C current density is achieved. This work represents an economical and feasible process to convert hazelnut shells into hard carbon.

## 1. Introduction

As a result of the growing industries and the irreversible depletion of fossil fuels, energy demand is tremendously increasing. Thus, the development of alternative energy sources, energy storage strategies and conversion devices such as fuel cell, capacitors and batteries are necessary. Among them, lithium-ion battery (LIB) technologies are widely used for mobile electronic devices and electric vehicles because of its high energy and power density and long cycle life [1–3]. The increasing demand of the battery market and depletion of lithium resources require alternative battery chemistries. Sodium-ion batteries (SIBs) have received much attention as one of the most promising candidates for electrical energy storage because of the abundance, wide availability of sodium resources, and chemical similarities of sodium to lithium [4,5].

However, the major challenge in commercializing sodium ion battery technology depends on finding suitable electrode materials, especially anode materials. Graphite is the most widely used anode material in LIBs due to its abundant resource, electronic conductivity, and cycling stability. However, ionic size of sodium ions is larger than lithium (1.02 Å of Na^+^ vs 0.76 Å of Li^+^) and it is not suitable for layer distance of graphite with conventional carbonate-based electrolytes [6,7]. Direct use of sodium metal as an anode causes some problems such as poor performance, easy short circuit, high chemical reactivity, and dendrite formation [8]. Up to now, anode materials with different ion storage mechanisms (based on the insertion, conversion and alloying reactions) have been researched by using carbon materials [9–11], transition metal oxides [12,13], alloys [14–16], or organic compounds [17–19]. Most of the anode materials (i.e. Sn, Sb or their oxides, sulfides, or alloys) used in the field of Na-ion batteries suffer from the large volume expansion during sodium ion insertion. On the other hand, organic compounds have insufficient cycling stability and low conductivity [6, 20]. Hard carbon is the most promising carbon anode material because of its large interlayer distance of the disordered carbon structure, and they can be prepared via simple conversion process of waste biomass [21,22].

In this study, we derived hard carbon material from hazelnut shell (HS) with two step carbonization processes: hydrothermal carbonization (HTC) and further carbonization at different temperatures. HS is a great energy potential of Turkey since it holds 75% of planted areas in the world [23]. The shells of hazelnut are important agricultural residues and represents approximately 50 wt.% of total hazelnuts. The structure of HS consists of cellulose and hemicellulose indicating that HS is a suitable carbon source to produce hard carbon [24]. Herein, we propose an efficient, low-cost, and simple method to synthesize hard carbon by using bio-waste. Firstly, HTC process was applied under mild reaction conditions and then further carbonization was applied at three different temperatures (500, 750, and 1000 °C). Finally, the resulting HS-derived hard carbon electrodes were performed as anode materials for sodium-ion battery.

## 2. Experimental

### 2.1. Materials

Hazelnut shell was collected from Trabzon, Turkey. Propylene carbonate (PC) (Sigma Aldrich): ethylene carbonate (EC) (Sigma Aldrich): sodium perchlorate (NaClO_4_) (Alfa Aesar): were used for electrolyte preparation. Polyvinylidene fluoride (PVdF) (Alfa Aesar) and carboxy methyl cellulose (CMC) (Doga Nanobiotech): Na-alginate (Alfa Aesar): polyacrilic acid (PAA) (Sigma Aldrich) were used as binders and 1-methyl-2-pyrrolidone (NMP) (Alfa Aesar) was used as a dissolving solvent of PVdF.

### 2.2. Synthesis of HS-derived hard carbon

HS-derived hard carbon anode material was synthesized with two different routes with/without HTC. For HTC, the collected shells were pulverized to obtain HS powder. Then, 0.5 g of HS powder, 18 mL distilled water and two drops of sulfuric acid (as a catalyst) were mixed. The mixture was transferred into Teflon-lined stainless-steel autoclave and synthesis was carried out under mild temperature (200 °C during 48 h rest time) in an oven. After HTC process, the sample was cooled down to room temperature and washed with pure water and ethanol, respectively. Resulting hydrochar sample was dried in an oven at 80 °C for 12 h.

**Figure 1:**
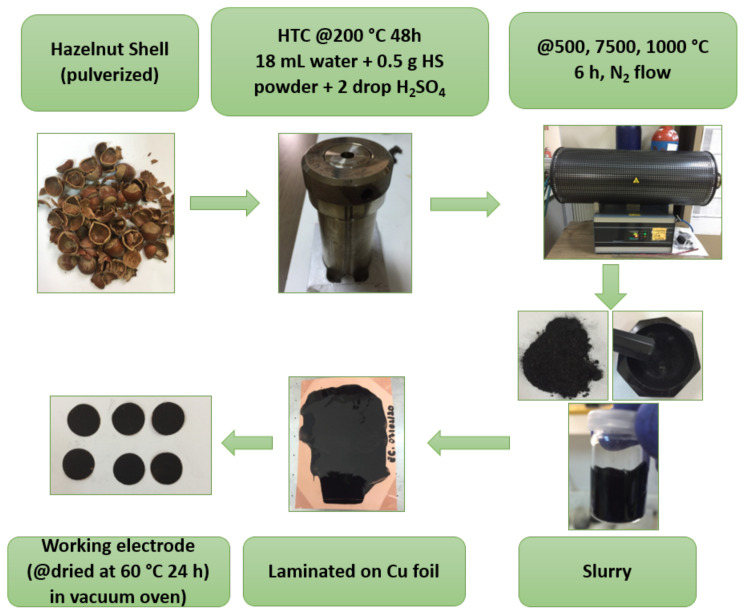
Schematic representation of the HS-derived hard carbon electrode.

Moreover, further carbonization process was performed to the hydrochar at various temperatures (500, 750, and 1000 °C) with heating rate of 2 °C min^−1^ under N_2_ atmosphere for 6 h in tubular furnace. Synthesized carbon materials were quoted as HS-HTC, HS-HTC@500, HS-HTC@750, HS-HTC@1000. For the sake of comparison, carbon materials without HTC process were also prepared. Direct carbonization of the HS powder was named as HS@500, HS@750, and HS@1000. The overall procedure is illustrated in Figure 1.

### 2.3. Physical characterizations

The thermal history and decomposition temperature of raw HS was characterized by thermogravimetric analysis (TGA) measurements (Perkin Elmer, 4000) performed at a heating rate of 10 °C min^−1^ between room temperature and 900 °C in an Argon atmosphere. The level of structural order of bare HS, hydrochar, and hard carbon were characterized by X-ray diffraction (XRD Bruker D8 Advance diffractometer (θ-2θ mode, Cu Kα radiation, λ = 1.5406): measurement was carried out in the range of 10–90 degrees and at a scanning speed of 2° min^−1^. The morphology of materials was investigated by scanning electron microscopy (SEM) (Philips XL30). Functional groups on bare HS and the resulting carbon materials were determined via Fourier-transform infrared spectroscopy (FTIR) spectroscopy (Perkin Elmer Spectrum 100).

### 2.4. Electrochemical measurements

The electrochemical performance of HS-derived electrodes was investigated by coin type cells assembled in an Argon filled glovebox. The working electrode was prepared by mixing active material with different binders (polyvinylidene fluoride (PVdF): carboxy methyl cellulose (CMC): Na-alginate, polyacrylic acid (PAA)), and super-P carbon at a weight ratio of 8:1:1. N-methyl-2-pyrrolidone (NMP, for PVdF binder) and distilled water (for CMC, Na-alginate, PAA binders) were used as dispersion mediums. Then, the slurry was laminated on copper foil and dried in a vacuum oven for 12 h. Na foil was used as both counter and reference electrodes. 1 M NaCIO_4_ ethylene carbonate/propylene carbonate was used as electrolyte. Galvanostatic charge/discharge measurements were carried out on the battery tester (Neware) at 0.005–2 V (vs Na/Na^+^) and 0.1C current density (1 C = 372 mAh × g^−1^). Electrochemical Impedance Spectroscopy (EIS) measurements were performed between the frequency range of 1 MHz and 10 kHz with voltage amplitude of 5 mV (VMP3 Bio-Logic). Cyclic voltammetry (CV) tests (Biologic VMP-3 electrochemical Workstation) were carried out in the voltage range of 0.005–2 V at various scan rates (0.2, 0.1, 0.05, 0.01 mV × s^−1^) to determine capacitive and diffusion controlled contribution.

## 3. Results and discussion

### 3.1. Material characterizations

**Figure 2:**
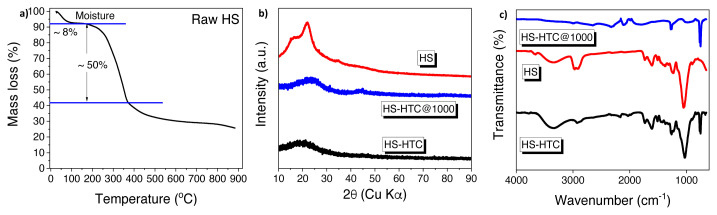
a) TGA data of raw HS at a heating rate of 10 °C min^−1^ under an Ar atmosphere b) XRD patterns of HS-HTC and HS-HTC@1000, c) FTIR spectra of bare HS, HS-HTC, and HS-HTC@1000.

There is a wide variety of biomass on earth and all of them have unique microstructure. These structural diversities directly affect the nature of the produced hard carbons. Ultimately, sodium ion diffusion and storage capacity depend on the structure of electrode materials [25]. Based on the analysis (wt.% of dry and ash free shell) hazelnut shell (HS) contains 50.8% of carbon, 42.6% of oxygen, 1.4% of nitrogen, and 5.2% hydrogen [26]. The amount of carbon contained in HS indicates that HS is a convenient raw material for the preparation of hard carbon materials. To investigate the thermal history and decomposition of raw HS, TGA was performed with temperature range from room temperature to 900 °C, as shown in Figure 2a. The TGA curve shows an initial mass loss of nearly 8% at 90 °C corresponding to moisture release. In addition, largest mass loss (50%) was observed at around 375 °C that could be attributed to the decomposition of cellulose/hemicellulose. On the other hand, lignin was decomposed in the temperature range of 175–600 °C and the significant mass loss was not observed when the temperature is higher than 600 °C. After all, the thermal decomposition process yield is 35.57%. To sum up, lignocellulosic materials are known to be decomposed above 200 °C [27,28] as we have also observed with the TGA measurement. Furthermore, the electrochemical performances of the resulting electrodes differ with the final carbonization temperatures [29,30]. Thus, in this research paper, we have carbonized the samples in three different temperatures and reported their electrochemical performances. Structural characterization and surface morphology of the materials were investigated with SEM, XRD, and FTIR measurements. XRD patterns of HS-derived hard carbon materials and hydrochar are shown in Figure 2b. HS-HTC@1000 has two broad peaks around 23º and 43º corresponding to (0 0 2) and (1 0 1) graphitic planes, respectively [31,32]. XRD patterns prove that the biomass-derived hard carbon is between amorphous and graphite (turbostratic) structures where graphene layers randomly sorted on each other. Intensity of the peaks increases with rising the carbonization temperature. Functional groups of bare HS, HS-HTC, and HS-HTC@1000 were examined by FTIR analysis (Figure 2c). The large absorption band seen at 3500–3000 cm^−1^ for HS and HS-HTC samples shows the presence of hydroxyl groups in the structure. HTC process has a step of aromatization, 750 cm^−1^ band can be attributed to the aromatic rings of the structure [33]. For bare HS and HS-HTC, the peak observed around 1690 cm^−1^ can be assigned to the C=O vibrations and these peaks were disappeared after carbonization process. Functional groups on surface can easily react with sodium ions and cause irreversible capacity losses [21].

**Figure 3:**
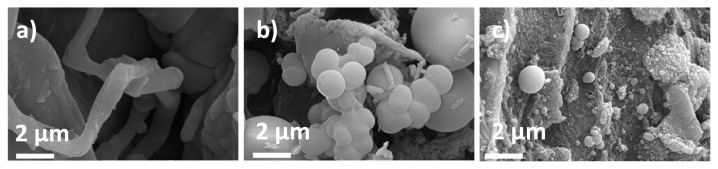
SEM images of a) bare HS, b) HS-HTC, c) HS-HTC@1000.

Depending on the SEM images of the bare HS, characteristic lignocellulosic structure, fibrous and sheet-like structures were observed (Figure 3). After the HTC process was applied, these fibrous structures began to be replaced by spherical microparticles since carbon formation through the HTC process relies on the emulsion polymerization like mechanism [21]. From the observations of SEM images, spherical microparticles transform into porous carbon structures by further increasing carbonization temperature to 1000 °C. Well-developed porous structure not only decreases the diffusion path of sodium ions but also enables electrolyte penetration into electrode.

Hydrochar contains functional group on its surface and has an amorphous structure. HTC process yields a low-grade of carbonization accompanied with the low degree of electrical conductivity. At this stage, cellulosic network does not completely degrade; thus, higher temperatures are needed [34,35]. Therefore, further carbonization process was applied to improve level of structural order.

### 3.2. Electrochemical characterizations

**Figure 4:**
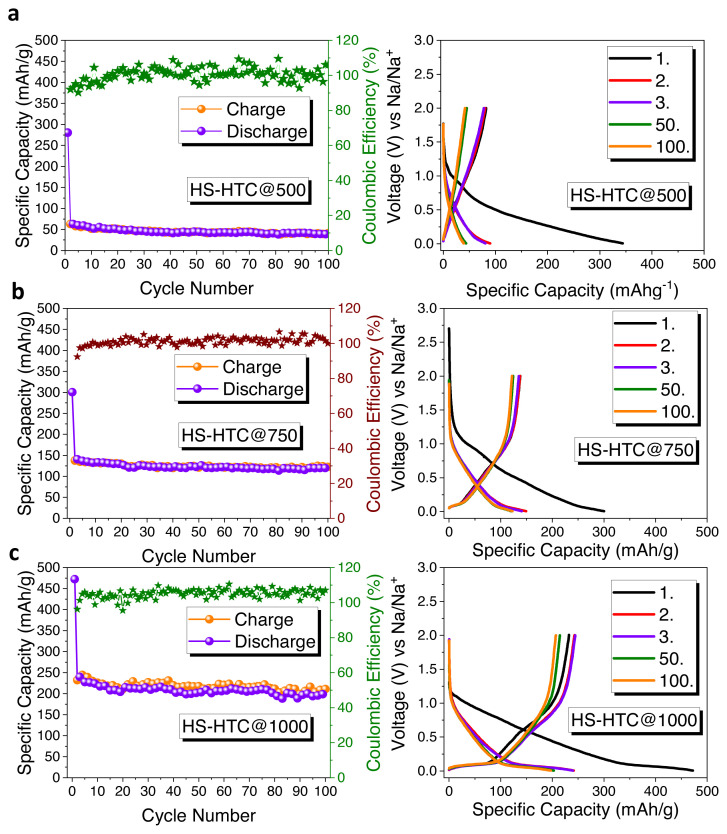
Cycle performances and galvanostatic charge/discharge curves of a) HS-HTC@500, b) HS-HTC@750, c) HS-HTC@1000 (Note: Electrodes were tested with PVdF binder).

The electrochemical characterizations of hard carbon electrodes were tested as anode materials for sodium ion battery by using half-cell configurations. Figure 4 represents the impact of different carbonization temperature on electrochemical performance of HS-derived hard carbon anode. Electrochemical performances were tested with galvanostatic charge/discharge cycling at 0.1C (1C = 372 mAh × g^−1^) current density, between 0.005 and 2 V vs Na/Na^+^. At the first cycle, charge/discharge capacities of HS-HTC@500, HS-HTC@750, and HS-HTC@1000 are 83/384 mAh × g^−1^ with an initial coulombic efficiency of 21.6%, 137/300 mAh × g^−1^ with an initial coulombic efficiency of 45.6% and 232/472 mAh × g^−1^ with an initial coulombic efficiency 49.1%, respectively. At the end of 100 cycles, HS-HTC@500 electrode delivered specific capacity of 54 mAh × g^−1^ with a capacity retention of 14%. HS-HTC@750 electrode delivered specific capacity of 122 mAh × g^−1^ with a capacity retention of 40.6% and HS-HTC@1000 electrode delivered specific capacity of 196 mAh × g^−1^ with a capacity retention of 41.5%.

The high irreversible capacity and low initial coulombic efficiency (ICE) are the results of the irreversibly intercalated sodium ions to the surface defects of hard carbon and the reaction between sodium ions and functional groups present on the surface. Solid electrolyte interphase (SEI) formation as a result of the decomposition of electrolyte can also cause the low ICE [36]. Thus, the sample treated at 1000 °C, HS-HTC@1000, has superior electrochemical performance that can be explained by its higher electrical conductivity.

**Figure 5:**
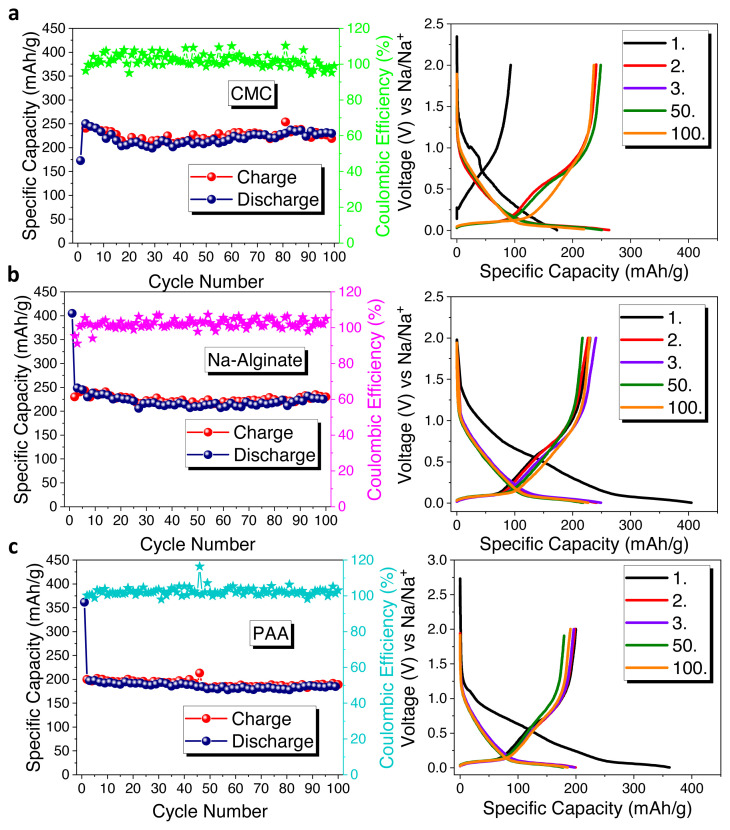
Cycle performances and galvanostatic charge/discharge curves of HS-HTC@1000 electrode with a) CMC, b) Na-alginate, and c) PAA binders.

Later, in order to study the binder effect on electrode performances, three different binders were tested with HS-HTC@1000 electrode shown in Figure 5. PVdF is the most widely used binder due to its good electrochemical performance. However, it was reported that the defluorination of PVdF can cause damage on hard carbon electrode [37]. As alternative binders, CMC, Na-alginate, and PAA can be suggested by their water-soluble character making them environmentally friendly in nature.

Initially, CMC binder was tested delivering the first charge/discharge capacities as 92/172 mAh × g^−1^ with an initial coulombic efficiency of 53.4% (Figure 5a). After 100 cycles, the electrode has 219 mAh × g^−1^ specific capacity. Then, Na-alginate binder was performed, and the electrode delivered around 230/404 mAh × g^−1^ charge/discharge capacities with a 55.6 % initial coulombic efficiency (Figure 5b). At the end of 100 cycles, the electrode demonstrated 232 mAh × g^−1^ specific capacity with a capacity retention of 57.4%. Lastly, PAA binder was performed, shown in Figure 5c, and the electrode delivered around 199/361 mAh × g^−1^ charge/discharge capacities with a 55.1% initial coulombic efficiency. Then the electrode yielded around 183 mAh × g^−1^ specific capacity with a capacity retention of 50.6% after 100 cycles. As a whole, when the binders’ impacts were compared, Na-alginate binder yielded a superior performance than that of the others. Na-alginate is a natural polysaccharide that contains carboxylic groups on each monomer units. CMC binder is cellulose derivative and produced synthetically, also has carboxylic groups on monomer units, but they are distributed randomly. These chemical differences between the two binders can explain the better electrode performance with Na-alginate binder. Uniform carboxylic groups can ensure stable SEI layer and homogeneous coverage [38]. Here, the resulting HS-based hard carbon electrodes were compared in terms of their electrochemical performances in Table 1. The effect of both hydrothermal carbonization and further carbonization temperature can be seen from the table. Among all samples that are investigated in this study, the composites synthesized at 1000 °C show the best results.

**Figure 6:**
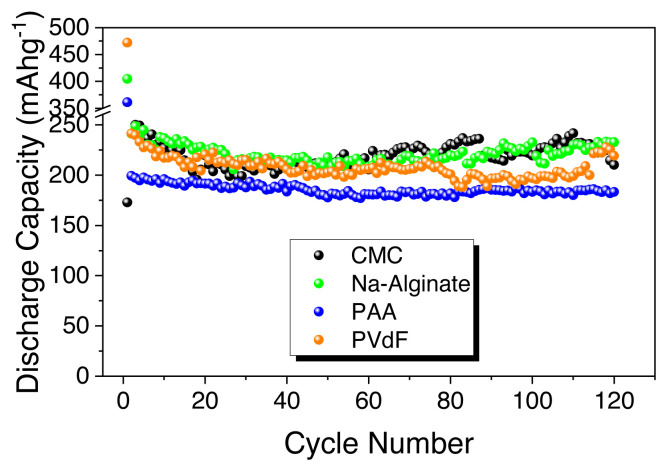
Discharge capacity comparison of HS-derived hard carbon electrode (HS-HTC@1000) with PVdF, CMC, Na-alginate, PAA binders.

Lastly, biomass-derived hard carbon electrodes reported in the literature as anodes for sodium ion batteries are shown in Table 2. Most of the hard carbons are produced from widely available agricultural waste, with a focus on elucidating the effect of carbonization processes and impact of binders. Thus, based on the comparison given in Table 2, it is clearly seen that HS-derived hard carbon can be promising anode materials for sodium ion batteries. To sum up, Figure 6 represents the comparison of the HS-HTC@1000 electrode with different binders emphasizing the best performance of the Na-alginate binder.

**Figure 7:**
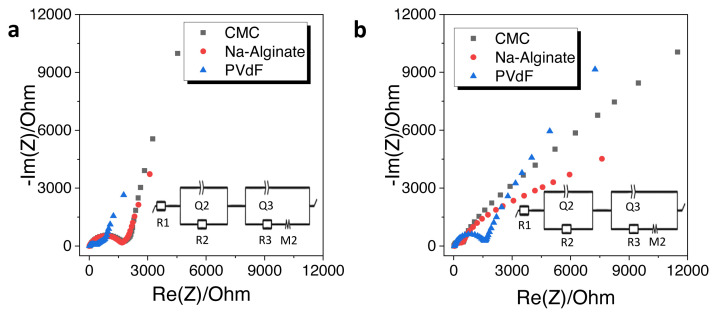
Electrochemical impedance spectra of HS-HTC@1000 with CMC, Na-alginate, and PVdF binders a) initial, b) after 10 charge/discharge cycles.

The impact of binders was further evaluated by impedance measurements. Figure 7 shows the Nyquist plots of HS-HTC@1000 electrode with CMC, Na-alginate and PVdF binders and the equivalent electric circuits were demonstrated as inset images. Impedance fitting parameters can be listed as the electrolyte resistance (R1): the charge transfer resistance (R2||Q2): the solid electrolyte interface resistance (R3||Q3): the restricted diffusion element (M) [39] and the results are shown in Table 3. All electrodes have one depressed semicircle in the high frequency and a straight line in low frequency. The semicircle at higher frequency is referred to the SEI formation on the surface and the charge transfer resistance at interface. Obviously, there is a difference between initial and after cycling resistances. PVdF showed the smaller semicircle diameter when compared with the other binders at the initial condition. From the observation of Figure 7 for PVdF binder, an increase of depressed-semicircle diameter was determined. Na-alginate and CMC binders have more stable SEI film formation. Moreover, defluorination of PVdF binder can be considered to additional interface and the side reactions can increase the resistance [6]. On the other hand, for CMC (R_initial_: 1829.7 ohm, R_total_: 318.4 ohm) and Na-alginate (R_initial_: 1671.8 ohm, R_total_: 514.3 ohm) binders, the initial resistance values were much higher than those after cycling. This was opposite for the PVdF binder, in which the initial total resistance was much lower than that of after cycling (R_initial_: 1621.7, ohm R_total_: 3058.4 ohm) supporting the defluorination of PVdF. Na-alginate and CMC binders have strong adhesion performance and uniform distribution on the electrode surface which ensure lower charge resistance. Smaller total resistance demonstrates that Na-alginate and CMC binders have more stable SEI layer [38, 40, 41]. Lower charge transfer resistance can ensure better cycling performance and kinetic properties of electrode [36].

**Figure 8:**
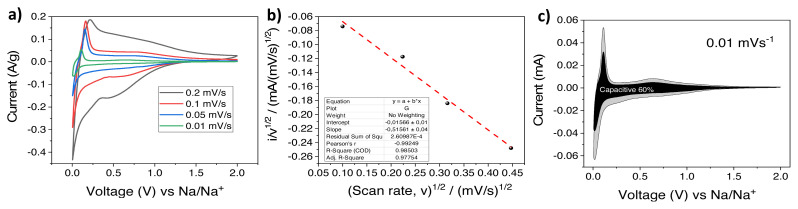
a) CV curves at various scan rates (0.2, 0.1, 0.05, 0.01 mV × s^−1^.) b) i/v ^1/2^ vs v ^1/2^ plot, c) capacitive contribution (black) and diffusion contribution (grey) at 0.01 mV × s^−1^.

CV analysis at different scan rates were also performed for the HS-HTC@1000 electrode at 0.2, 0.1, 0.05, 0.01 mV × s^−1^ (Figure 8a). Equation (1) was used to be able to calculate the capacitive and diffusion-controlled contributions.


(1) $$\text{i}(\text{V})={\text{k}}_{1}\text{v}+{\text{k}}_{2}{\text{v}}^{1/2}$$

At a fixed potential, k_1_v represents capacitive current, k_2_v^1/2^ indicates diffusion controlled current. When equation (1) is rearranged (scan rate: ν, constant: k_1_and k_2_) and plotted versus i/v^1/2^ to v^1/2^, k_1_ refers to slope and k_2_ is the intercept (Figure 8b) [42,43]. Then, the contribution ratios were analyzed for all sweep rates by peaking up the voltage values from 0.2 to 1.0 V (vs Na) at every 0.1 interval. Figure 8c demonstrates the results from the scan rate of 0.01 mV × s^−1^ in which the capacitive contribution of the total capacity is 60%. As expected, the proportion of capacitive contribution increased with the scan rate (60%, 77%, 82% where the scan rates are 0.01, 0.05, 0.1 mV × s^−1^, respectively).

## 4. Conclusions

In summary, we have effectively synthesized hazelnut shell-derived low-cost hard carbons by using hydrothermal treatment and further carbonization at different temperatures. After further carbonization process, all the samples have layer distances larger than the graphite which is suitable for sodium ion storage. Hard carbon prepared at 1000 °C with sodium alginate binder yielded superior electrochemical performance as anode for sodium ion battery with a reversible capacity of 232 mAh × g^−1^ at 0.1 C current density. This promising performance along with the simple, economical, and scalable synthesis process can make biomass-derived hard carbon a promising anode material for sodium ion batteries. Besides, water-soluble binders show that environmentally friendly binders can be potential candidates for conventional PVdF binder.

## Figures and Tables

**Table 1:** Cycle performances of electrodes.

Electrode	Binder	Cycle number	Capacity (mAh × g^−1^) discharge-charge
HS@500	PVdF	1	280–63
		100	41–39
HS@750	PVdF	1	321–121
		100	48–61
HS@1000	PVdF	1	283–93
		100	101–100
HS-HTC@500	PVdF	1	344–83
		100	54–53
HS-HTC@750	PVdF	1	300–137
		100	122–122
HS-HTC@1000	PVdF	1	472–232
		100	196–210
HS-HTC@1000	CMC	1	172–92
		100	219–236
HS-HTC@1000	Na-alginate	1	404–230
		100	232–231
HS-HTC@1000	PAA	1	361–199
		100	183–190

**Table 2:** Biomass-derived hard carbon anodes for sodium ion batteries.

Biomass precursor	Reversible capacity	Current density	Ref.
Lotus stem	250 mAh × g^−^^1^	100 mA × g^−^^1^	[44]
Rice husk	276 mAh × g^−^^1^	0.1 C	[45]
Grass	200 mAh × g^−^^1^	100 mA × g^−^^1^	[46]
Mangosteen shell	330 mAh × g^−^^1^	20 mA × g^−^^1^	[47]
Apricot shell	184 mAh × g^−^^1^	0.1 C	[21]
Rambutan peel	225 mAh × g^−^^1^	0.1 C	[22]
Tea leaves	179 mAh × g^−^^1^	100 mA × g^−^^1^	[48]
Macadamia nutshell	220 mAh × g^−^^1^	20 mA × g^−^^1^	[49]
Unburned charcoal	292.3 mAh × g^−^^1^	50 mA × g^−^^1^	[50]
Hazelnut shell	232 mAh × g^−^^1^	0.1 C	this study

**Table 3:** Impedance fitting parameters.

	CMC	Na-alginate	PVdF
Before cycles			
R_1_ (Ω)	33.24	9.523	5.579
R_2_ (Ω)	1660	1660	322.2
R_3_ (Ω)	136.5	2.289	1294
After cycles			
R_1_ (Ω)	17.97	37.39	35.47
R_2_ (Ω)	297	476.8	1590
R_3_ (Ω)	3.455	0.11	1433
